# Platelets and platelet adhesion molecules: novel mechanisms of thrombosis and anti-thrombotic therapies

**DOI:** 10.1186/s12959-016-0100-6

**Published:** 2016-10-04

**Authors:** Xiaohong Ruby Xu, Naadiya Carrim, Miguel Antonio Dias Neves, Thomas McKeown, Tyler W. Stratton, Rodrigo Matos Pinto Coelho, Xi Lei, Pingguo Chen, Jianhua Xu, Xiangrong Dai, Benjamin Xiaoyi Li, Heyu Ni

**Affiliations:** 1 Department of Laboratory Medicine and Pathobiology, University of Toronto, Toronto, ON Canada; 2 Department of Laboratory Medicine, Keenan Research Centre for Biomedical Science, St. Michael’s Hospital, Toronto, ON Canada; 3 Guangdong Provincial Hospital of Chinese Medicine, Guangzhou University of Chinese Medicine, Guangzhou, Guangdong People’s Republic of China; 4 Canadian Blood Services, Toronto, ON Canada; 5 CCOA Therapeutics Inc, Toronto, ON Canada; 6 Lee’s Pharmaceutical holdings limited, Shatin Hong Kong, China; 7 Zhaoke Pharmaceutical co. limited, Hefei, Anhui China; 8 Hong Kong University of Science and technology, Hong Kong, China; 9 Department of Medicine and Department of Physiology, University of Toronto, Toronto, ON Canada

**Keywords:** αIIbβ3, Anfibatide, GPIbα, GPVI, Hemostasis, Integrins, P-selectin, Stroke, Thrombosis

## Abstract

Platelets are central mediators of thrombosis and hemostasis. At the site of vascular injury, platelet accumulation (i.e. adhesion and aggregation) constitutes the first wave of hemostasis. Blood coagulation, initiated by the coagulation cascades, is the second wave of thrombin generation and enhance phosphatidylserine exposure, can markedly potentiate cell-based thrombin generation and enhance blood coagulation. Recently, deposition of plasma fibronectin and other proteins onto the injured vessel wall has been identified as a new “protein wave of hemostasis” that occurs prior to platelet accumulation (i.e. the classical first wave of hemostasis). These three waves of hemostasis, in the event of atherosclerotic plaque rupture, may turn pathogenic, and cause uncontrolled vessel occlusion and thrombotic disorders (e.g. heart attack and stroke). Current anti-platelet therapies have significantly reduced cardiovascular mortality, however, on-treatment thrombotic events, thrombocytopenia, and bleeding complications are still major concerns that continue to motivate innovation and drive therapeutic advances. Emerging evidence has brought platelet adhesion molecules back into the spotlight as targets for the development of novel anti-thrombotic agents. These potential antiplatelet targets mainly include the platelet receptors glycoprotein (GP) Ib-IX-V complex, β3 integrins (αIIb subunit and PSI domain of β3 subunit) and GPVI. Numerous efforts have been made aiming to balance the efficacy of inhibiting thrombosis without compromising hemostasis. This mini-review will update the mechanisms of thrombosis and the current state of antiplatelet therapies, and will focus on platelet adhesion molecules and the novel anti-thrombotic therapies that target them.

## Background

Platelet adhesion, activation and aggregation are critical events in hemostasis and thrombosis [1–3]. Platelet adhesion molecules, αIIbβ3 integrin and the glycoprotein (GP) Ib-IX-V, are essential for these processes [4–6]. Other adhesion molecules, such as P-selectin, GPVI and cadherins, are also involved [7–10]. The important roles of adhesion molecules in normal hemostasis have been well demonstrated in bleeding disorders, for example, Glanzmann thrombasthenia (β3 integrin deficiency) [11] and Bernard-Soulier syndrome (GPIb-IX-V complex deficiency) [12]. However, under pathological conditions, excessive platelet function may lead to thrombotic diseases, such as myocardial infarction and ischemic stroke, which cause far more deaths each year than cancer or respiratory diseases [1, 2, 13–15]. Therefore, antiplatelet agents are vital for the treatment of thrombosis [16]. For over a decade, dual antiplatelet therapy with clopidogrel and aspirin has been considered a key treatment of patients with acute coronary syndrome [17, 18]. Nonetheless, some patients undergoing this combination therapy continue to suffer from recurrent thrombotic events, likely a result of platelet activation and aggregation occurring independently of ADP or thromboxane A2 receptor-mediated signalling pathways [17]. Thus, attenuating platelet adhesion appears to be a desirable strategy in effectively controlling pathological thrombosis [18]. Further understanding of the interactions between platelet adhesion molecules and their binding partners is therefore crucial in developing novel anti-thrombotic therapies. This review briefly summarizes the current knowledge of thrombosis and antiplatelet therapies, introduces a number of major platelet adhesion molecules, and highlights some recent advances in the new mechanisms of thrombosis, and anti-thrombotic therapies that are in clinical trials (unless otherwise indicated). There are several excellent available reviews regarding antiplatelet therapies, such as ADP antagonists (e.g. P2Y12 inhibitors), thromboxane antagonists and PAR-1/4 inhibitors [17, 18]. This mini-review will mainly focus on the therapeutic developments targeting platelet adhesion molecules.

## Review

### Arterial thrombosis and current state of antiplatelet therapies

Arterial thrombosis at the site of atherosclerotic plaque rupture may lead to uncontrolled vessel occlusion, resulting in life-threatening consequences (e.g. unstable angina, myocardial infarction and ischemic stroke) [1, 2, 13]. During plaque rupture, subendothelial matrix proteins, like collagen, von Willebrand factor (VWF), fibrinogen, fibronectin and laminin are exposed to circulation, leading to the rapid response of platelets [6]. Inappropriate platelet adhesion, activation and aggregation promote excessive platelet plug formation. Activated platelets can also provide negatively-charged surfaces that harbor coagulation factors and markedly potentiate cell-based thrombin generation and blood coagulation [1, 2, 19, 20]. The evolving concept of the “protein wave of hemostasis” indicates a potential role of platelet-released plasma fibronectin in thrombosis and hemostasis [21, 22]. Thus, platelets are key mediators of atherothrombosis, which are actively involved in all three waves of thrombus formation: protein wave, platelet accumulation, and blood coagulation [21, 23].

**Fig. 1:**
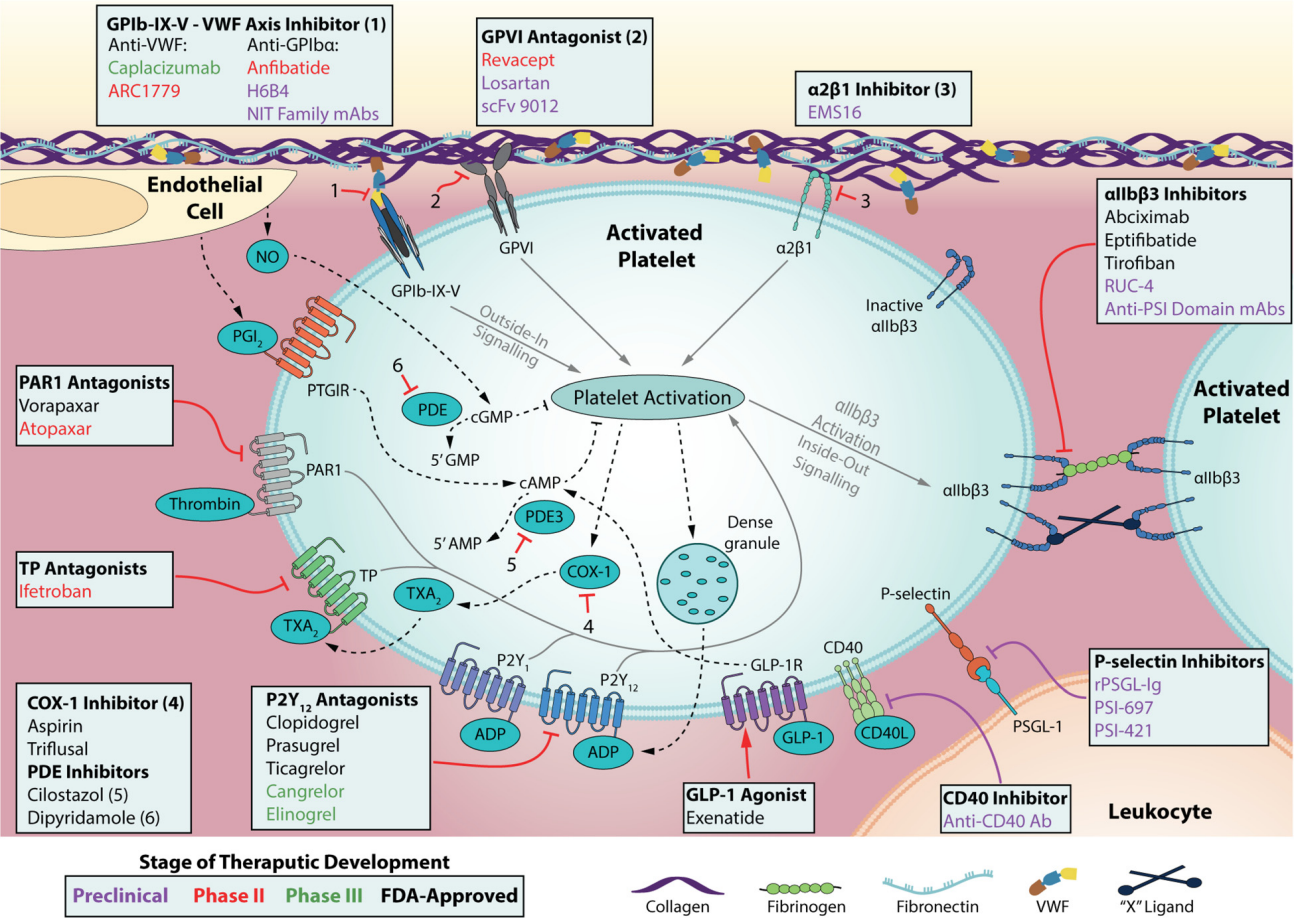
Current and novel antiplatlet therapies. Platelet adhesion to an injury site at a vessel wall is mediated by the exposure and binding of subendothelial matrix proteins (e.g. collagen, VWF, fibrinogen, and fibronectin) to glycoprotein (GP) receptors on the platelet surface. VWF binding to the GPIb-IX-V complex, collagen binding to platelet GPVI and integrin α2β1 receptors trigger a signal transduction process resulting in the local release of platelet activation agonists, such as thromboxane A2 and ADP. These agonists along with thrombin produced from coagulation cascades and activated platelets, bind to platelet surface bound G-coupled receptors inducing further platelet activation. Activation of platelet integrin αIIbβ3 induces platelet aggregation mediated by fibrinogen/VWF or the yet undetermined “X” ligands. Leukocyte-platelet adhesion can be driven by the interaction between platelet surface P-selectin and its counter-receptor PSGL-1 situated upon the leukocyte surface. Inhibition of platelet activation is mainly mediated by the PDE/PDE3 regulated degradation and PGI_2_, NO and GLP-1R regulated activation of cGMP or cAMP. Direct and indirect antithrombotic therapeutics are tabulated in the light colored boxes within the figure. The actions of antithrombotic therapies are depicted using *red* arrows, and some indirect antithrombotic agents (such as anti-atherosclerotic agents) are represented with *purple* arrows. Therapeutics, to name a few, listed in *black*, *green*, *red* and *purple* correspond to FDA-approved, phase III, phase II or preclinical development status, respectively. Numbered inhibitory arrows represent the actions of the correspondingly numbered therapies. Some other anti-platelet agents are not included, more information can be found in references 17, 18 and other publications. *Abbreviations*: *COX-1* cyclooxygenase 1 *GLP-1* glucagon-like peptide 1, *GLP-1R* glucagon-like peptide 1 receptor, *PAR* protease-activated receptor, *PDE* phosphodiesterase, *PSGL-1* P-selectin glycoprotein ligand 1, *TP* thromboxane prostanoid receptor, *TXA*_*2*_ thromboxane A2; *VWF* von Willebrand factor

Current FDA-approved antiplatelet therapies (Fig. 1) mainly aim to (i) inhibit thromboxane A2 synthesis, which inhibits platelet activation (e.g. aspirin and triflusal); (ii) antagonize the function of platelet P2Y12 receptors, (e.g. clopidogrel, prasugrel, and ticagrelor); (iii) inhibit platelet integrin αIIbβ3 activity, which inhibits platelet aggregation, (e.g. abciximab, eptifibatide, and tirofiban); (iv) inhibit phosphodiesterase, which increases platelet cAMP/cGMP levels (e.g. dipyridamole and cilostazol) [24]. These antiplatelet drugs have significantly reduced cardiovascular deaths. However, limitations of current therapies, such as weak/poor inhibition of platelet function, excessive bleeding, thrombocytopenia and unexpected platelet activation are concerns that drive therapeutic advances [18, 25, 26]. In 2014, the FDA approved Vorapaxar, a novel antagonist of the thrombin receptor protease-activated receptor 1 (PAR1), which reduces the risk of heart attack and stroke in patients with atherosclerosis or peripheral arterial disease [27, 28]. However, Vorapaxar must not be used in patients who have histories of stroke, transient ischemic attack (TIA) or intracranial hemorrhage, since it increases the risk of intracranial bleeding [28, 29].

### Platelet adhesion molecules in hemostasis and thrombosis: novel mechanisms and therapeutic opportunities

Platelet adhesion molecules are proteins/receptors on the platelet surface that interact with other cells or the extracellular matrix, including the integrin family (e.g. α2β1, α5β1, α6β1, αLβ2, αIIbβ3, and ανβ3) [4, 30, 31], the immunoglobulin superfamily (e.g. GPVI, FcγRIIA, ICAM-2, PECAM-1, JAMs and Cadherin 6), the leucine-rich repeat family (LRR; e.g. GPIb-IX-V complex), and the C-type lectin receptor family (e.g. P-selectin and CLEC-2), etc. [32–34]. Recent evidence has shown that platelet adhesion molecules play key roles in a variety of pathophysiological processes [23], such as hemostasis and thrombosis [4, 33], immune responses [35, 36], inflammation [35–37], atherosclerosis [38–40], lymphatic vessel development [41–44], angiogenesis [45–47], miscarriage [48, 49], and tumor metastasis [50–52]. Platelets are versatile cells and the mechanisms of their diverse functions have emerged as hot research topics [23]. This review mainly focuses on their roles in thrombosis and as novel anti-thrombotic targets (Fig. 1).

#### The GPIb-IX-V complex: emerging targets of antiplatelet therapy

##### New insights into the GPIb-IX-V complex

Platelet GPIb-IX-V complex (LRR family protein) has approximately 50,000 copies/platelet. It is composed of one GPIbα subunit disulfide-linked to two molecules of GPIbβ, and non-covalently linked with GPIX and GPV in a 2:4:2:1 ratio [53]. GPIb-IX-V is a key platelet receptor in initiating platelet translocation and adhesion to the vessel wall during vascular injury, especially under high shear stress (e.g. in small or stenosed arteries) [54, 55]. Platelet translocation onto the subendothelium is mediated by the binding of GPIbα to the immobilized VWF, a multimeric adhesive protein secreted from activated endothelial cells and platelets. The crystal structure of the GPIbα N-terminal ligand-binding domain and the VWF A1 domain gives useful information regarding their interaction [56]. This interaction induces intracellular signalling events that can activate integrins, leading to platelet stable adhesion and subsequent platelet aggregation. Interestingly, platelet-derived VWF was recently shown not essential for hemostasis and thrombosis, but instead fosters thrombo-inflammatory diseases such as ischemic stroke in mice via a GPIb-dependent mechanism [57]. This suggests that targeting GPIbα-VWF may be a promising anti-thrombotic strategy, particularly in thrombo-inflammatory conditions.

Furthermore, GPIb-IX-V complex has a high affinity for thrombin [58, 59]. Two thrombin binding sites on GPIbα LRR C-terminal flank region have been revealed [58]. Consequently, thrombin can activate platelets via GPIbα in two ways [60]: accelerating the cleavage of PAR-1 and platelet activation [61], or direct signaling via GPIbα, particularly after cleaving GPV, which is generally considered a “brake” in GPIb-IX-V activation [62, 63]. It is currently unknown but it is reasonable to consider that targeting both VWF and thrombin binding sites of GPIbα might provide additional benefits in effectively controlling thrombosis.

GPIbα can also interact with multiple other ligands, leading to platelet activation (e.g. thrombospondin [64] and P-selectin), pro-coagulant activity (e.g. factors XI [65], XII [66], VIIa [67] and kininogen [68]), inflammatory responses (e.g. P-selectin [69, 70], α_M_β_2_ [71]), arterial remodeling [72] and others. Recently, the antibody-GPIbα interaction in immune thrombocytopenia has been highlighted. Some anti-GPIbα antibodies cause platelet activation and desialylation (removal of sugars), followed by the clearance of desialylated platelets via Ashwell-Morell receptors on hepatocytes [73, 74].

##### Developing novel antiplatelet agents against GPIbα

Given the critical roles of GPIbα or GPIbα-VWF interactions in platelet adhesion, particularly under stenosis high-shear conditions, they are attractive targets in attenuating thrombosis [54, 75, 76]. Currently, two such agents are in active clinical trials. ALX-0081 (Caplacizumab), an anti-VWF humanized single-variable-domain immunoglobulin (Nanobody), binds to the A1 domains of VWF with high affinity [77]. The phase I and II clinical trials of ALX-0081 in patients with stable angina undergoing percutaneous coronary intervention (PCI) or high risk PCI patients have shown a promising antiplatelet effects, and a relatively safe profile [77, 78]. The phase III clinical trials will investigate its effects on acquired thrombotic thrombocytopenic purpura (TTP) [79–81]. ARC1779, an anti-VWF aptamer, was previously reported as an encouraging agent; however, the clinical trial of ARC1779 was prematurely terminated [82]. These VWF inhibitors may be useful candidates for TTP treatment.

A direct anti-GPIbα drug, Anfibatide, is purified from the snake venom of *Agkistrodon acutus* [83, 84]. Notably, Anfibatide inhibits both VWF and α-thrombin binding to GPIbα, representing a more potent anti-thrombotic effect [85]. In experimental models, Anfibatide inhibited platelet adhesion, aggregation and thrombus formation, without increasing bleeding time [83]. The phase II human clinical trials have also shown the promise of Anfibatide being utilized as a novel antiplatelet agent in cardiovascular diseases without significantly affecting hemostasis in patients with non-ST segment elevation myocardial infarction (unpublished data) [85]. Additionally, anti-GPIbα antibody displayed a strong protective effect in the mouse stroke models without inducing significant intracranial bleeding [86–88]. Anfibatide has also been shown as a candidate to treat ischemic stroke in experimental models [89] (the same may hold true for anti-VWF therapy) and deserves further investigation. There are some other preclinical agents targeting GPIbα that are under investigation, such as h6B4-Fab [90], GPG-290 [91], and anti-GPIbα NIT family monoclonal antibodies [92]. The generation of these novel antagonists is reaching the forefront of treatment against heart attack and stroke, although the efficacy and safety of these drugs remain to be further established or evaluated in human clinical trials. Notably, there are currently no clinically available direct GPIbα antagonists.

#### GPVI: a potential anti-thrombotic target

GPVI (immunoglobulin superfamily protein) is exclusively expressed on platelets and megakaryocytes. It is associated with the Fc receptor γ-chain, which contains an immunoreceptor tyrosine-based activation motif (ITAM). Cross-linking by ligands, such as collagen, leads to ITAM-dependent signalling, and platelet activation. A possible anti-thrombotic benefit of targeting PI3-kinase/Akt pathway on ITAM receptors was suggested [93]. Fibrin has also been identified as a new GPVI ligand [94]. The GPVI ectodomain interacts with immobilized fibrin, which amplifies thrombin generation, and promotes thrombus stabilization [94, 95].

The role of platelet GPVI in the pathogenesis of ischemic stroke has been gradually acknowledged [96–98]. Notably, platelet adhesion/activation can enhance infarct growth by promoting an inflammatory response [88, 99, 100]. GPVI-mediated platelet activation can lead to the release of interleukin-1α that drives cerebrovascular inflammation [100]. GPVI may be thus a potential antiplatelet target [97, 101, 102]. In animal models, anti-GPVI protected against thrombosis, ischemia-reperfusion injury [103] and stroke [104]. In phase I clinical trials, Revacept (the humanized Fc fusion protein of the GPVI ectodomain), inhibited collagen-induced human platelet aggregation [105]. Phase II trials of Revacept in patients with carotid artery stenosis, TIA, or stroke are ongoing [106]. The efficacy and safety of Revacept in these patients will be further determined. Some other GPVI targeted agents that are under preclinical development, such as Losartan [107] and scFv9012 [108], have been shown to inhibit the binding of GPVI to collagen.

#### Platelet integrin receptors

Integrins are heterodimeric transmembrane receptors, which are involved in cell-cell and cell-matrix interactions [30]. There are six different integrins on platelet surfaces: α2β1, α5β1, α6β1, αLβ2, αIIbβ3, and ανβ3. Platelet integrin αIIbβ3 is the dominant integrin expressed on platelets. Given the critical roles of αIIbβ3 integrin in mediating platelet aggregation, αIIbβ3 antagonists have been widely used for nearly two decades.

##### Integrin αIIbβ3 as anti-thrombotic targets: lessons and opportunities

Approximately 17 % of total platelet surface proteins are αIIbβ3 integrin, which contains both αIIb and β3 subunits [4]. Platelet “outside-in” signals are induced following platelet adhesion and platelet activation (e.g. GPIbα-VWF, GPVI/α2β1-collagen, P2Y_12_-ADP, PARs-thrombin), resulting in an increased Ca^2+^ influx and ultimately “inside-out” signaling. These “inside-out” signals further drive the conformational changes of αIIbβ3, from a low to high affinity state for binding to its ligands (e.g. fibrinogen/fibrin, VWF, fibronectin, thrombospondin, vitronectin and unidentified “X” ligands) [109–112].

Fibrinogen, a major prothrombotic ligand of αIIbβ3, has been documented to be required for platelet aggregation for over 50 years. However, platelet aggregation still occurs in the absence of fibrinogen and VWF, although in the absence of αIIbβ3, aggregation is abolished [5, 8, 21, 113–116]. The discovery of “fibrinogen-independent platelet aggregation” demonstrates that unidentified αIIbβ3 ligands also mediate platelet aggregation [5, 8, 21, 113, 116], and have the potential to be novel anti-thrombotic targets. Interestingly, some ligands (e.g. plasma fibronectin, vitronectin) may block prothrombotic ligand (e.g. fibrinogen)-αIIbβ3 interactions and attenuate thrombosis [21, 117].

Three FDA-approved αIIbβ3 antagonists are available: Abciximab (ReoPro), Eptifibatide (Integrilin) and Tirofiban (Aggrastat) [118–120]. Abciximab is a fragmented antibody that binds close to the ligand binding-pocket on αIIbβ3. Eptifibatide, isolated from snake venom, binds via a KGD sequence and is a competitive inhibitor for fibrinogen-αIIbβ3, whilst tirofiban is a small molecule RGD inhibitor. Currently, αIIbβ3 antagonists are used in patients undergoing PCI and significantly decrease the incidence of myocardial infarction and death [121]. However, these antagonists can induce further conformational changes in the β3 subunit that may have negative consequences, such as exposing previously hidden epitopes, and causing platelet activation [122]. αIIbβ3 antagonists are also associated with intracranial hemorrhage in patients with acute ischemic stroke [123]. Therefore, a safer and more specific on-target drug is required to provide better patient care. Recently, a novel αIIbβ3 antagonist, RUC-4 (a more potent and more soluble congener of RUC-2 that disrupts Mg^2+^ binding to the metal ion-dependent adhesion site of αIIbβ3), is suggested for prehospital therapy of myocardial infarction in animal models, without significantly priming the receptor to bind fibrinogen [124]. However, the possibility of increased bleeding with therapeutic doses of RUC-4 remains to be evaluated [124].

The plexin-semaphorin-integrin (PSI) domain, located near the N-terminus of the β3 subunit, is highly conserved across the integrin family in different species, and contains seven cysteine residues which have been implicated in regulating β2 integrin activation [125, 126]. Previous studies described a role for cysteine-derived thiol/disulfide groups in the conformational switches of the β3 integrin [127–130]. Disulfide bond remodeling is mediated primarily by thiol isomerase enzymatic activity, which is derived from active CXXC thioredoxin motifs and plays a role in the activation of αIIbβ3 [131]. Our group has recently identified that integrin PSI domain has endogenous thiol isomerase function and could be a novel target for anti-thrombotic therapy (unpublished data) [132]. We found that both CXXC motifs of β3 integrin PSI domain are required to maintain the optimal enzyme function, since mutations to one or both of the CXXC motifs decrease or abolish their protein disulphide isomerase (PDI)-like activity. We developed anti-PSI monoclonal antibodies and found that these antibodies cross-reacted with β3 PSI domains of human and other species and specifically inhibited the PDI-like activity, integrin activation and reduced PAC-1 binding to β3 integrin. Importantly, anti-PSI abrogated murine and human platelet aggregation in vitro and thrombus growth ex vivo and in vivo in both small and large vessels without significantly affecting bleeding time or platelet count. Thus, integrin PSI domain contains endogenous PDI activity and is a key regulator of integrin activation that can be a new target for therapy.

Interestingly, targeting activated platelets αIIbβ3 has been considered into the development of novel fibrinolytic drugs, which may allow effective thrombolysis and thromboprophylaxis [14, 133]. For example, scFvSCE5 (a single-chain urokinase plasminogen activator fused to a small recombinant antibody that binds activated αIIbβ3) directly targets thrombi and exerts an effective thrombolysis [133]. A chimeric platelet-targeted urokinase prodrug (composed of a single-chain version of the variable region of an anti-αIIbβ3 mAb and a thrombin-activatable, low-molecular-weight pro-uPA) selectively targets new thrombus formation [134].

##### Other platelet integrins: α2β1, α6β1 and α5β1 

Other integrin receptors may also be considered as novel anti-thrombotic targets [16, 135]. Platelet α2β1 promotes stable platelet adhesion to collagen and may be a viable option, since overexpression of α2β1 in humans increases atherothrombotic risk, but lower level of α2β1 does not enhance bleeding risk [16]. Experimental evidence shows that α2β1 inhibitors (e.g. snake venom EMS-16) reduced pathological thrombus formation in vivo [136–138]. Platelet α6β1, the main receptor for laminin, plays a role in platelet adhesion/activation and arterial thrombosis, and may also be a new target [135]. Platelet α5β1, the major receptor for fibronectin, plays a supplementary role in platelet adhesion [139], but evidence is lacking regarding the anti-thrombotic benefits of antagonizing α5β1.

#### Other novel anti-thrombotic candidates: Glucagon-like peptide 1 receptor, P-selectin, CD40/CD40L, and Toll-like receptors

Strategies to target other platelet receptors beyond adhesive proteins have also been developed, such as P2Y12, PAR1, TP, 5HT_2A_ antagonists [17, 140]. Interestingly, some chronic diseases, such as diabetes mellitus and atherosclerosis, are associated with arterial thrombosis [23, 141]. Recently, our group identified that a functional Glucagon-like peptide 1 receptor (GLP-1R) is expressed on human megakaryocytes and platelets [142]. Importantly, GLP-1R agonists (e.g. Exenatide), likely through increasing the intracellular cAMP levels, inhibit platelet function and thrombus formation [142]. This study provides important insights into why diabetic patients who are receiving GLP-1-targeted therapies have a reduced number of cardiovascular events [142, 143]. In addition, given the cross-talks between platelets and immune systems, thrombosis also intensively communicates with the inflammatory pathway [23]. Some anti-inflammatory/anti-atherosclerotic agents may therefore also indirectly inhibit thrombosis, especially in deep vein thrombosis [144]. For example, antagonists of P-selectin/PSGL-1, such as rPSGL-Ig [145], PSI-697 [146], PSI-421 [147], inhibit platelet-mediated leukocyte attachment and recruitment of procoagulant microparticles, and may represent a safe therapeutic intervention in accelerating thrombolysis [148]. Antagonists of CD40/CD40L [149], such as CD40 antibody, reduce atherosclerotic burden in a murine model [150]. In addition, as the important roles of Toll-like receptors in atherosclerosis are gradually recognized [151, 152], they may also be potential targets for the treatment of atherothrombosis.

## Conclusions

Arterial thrombotic events, such as myocardial infarction and ischemic stroke, and venous thromboembolism, are three leading causes of morbidity and mortality worldwide [153]. Platelets play a central role in the pathogenesis of atherothrombosis, and contribute profoundly to the pathology of venous thrombosis [23]. Platelet adhesion molecules, act as the contacts between platelets and other cells or extracellular matrix proteins and, to a great extent, may determine the reactivity of platelets and thus are attractive anti-thrombotic targets (Fig. 1) [23]. Although evidence-based antiplatelet therapy has markedly improved patient care, on-treatment events and bleeding are still major concerns [17, 148].

Optimization of the use of currently available therapies, and improvements to the understanding of individual differences in response to anti-platelet treatments are still the most cost-effective treatment strategies [17, 148]. Additionally, improved understanding of the mechanisms of platelet accumulation has been critical for further developing novel antiplatelet therapies, such as the PAR1 antagonist Vorapaxar (recently approved by the FDA), GPIbα/VWF antagonists (e.g. ALX-0081 and Anfibatide; undergoing clinical trials), and GPVI antagonist (e.g. Revacept; undergoing clinical trials) (See section II. A-C). Another cost-effective strategy may be to repurpose already-established drugs by discovering novel mechanisms of action in anti-thrombotic diseases, such as the recently-identified GLP-1R agonist, Exenatide, an anti-diabetic drug that has potential anti-thrombotic effects [142, 154]. Future studies in the areas of atherothrombosis, inflammation, metabolic syndrome, diabetes, lipid metabolism and cancer-related thrombotic diseases in the next few years should advance our knowledge and the application of these and other new anti-platelet agents. Of note, clinical trials provide important evidence regarding the safety and efficacy of the treatments. However, difficulties such as narrow eligibility criteria, low enrollment of patients and the necessity to test the new drugs on top of the current dual antiplatelet therapy (e.g. aspirin and clopidogrel), may add﻿ complexity to the development of new drugs and also deserve our attention.

## Abbreviations

ADP: Adenosine diphosphate
GLP-1R: Glucagon-like peptide 1 receptor
GP: Glycoprotein
ITAM: Immunoreceptor tyrosine-based activation motif
ITP: Idiopathic thrombocytopenic purpura
LLR: Leucine-rich repeat
PAR: Protease-activated receptor
PCI: Percutaneous coronary intervention
PDI: Protein disulphide isomerase
PSI: Plexin-semaphorin-integrin
TIA: Transient ischemic attack
TTP: Thrombotic thrombocytopenic purpura
VWF: von Willebrand factor
